# The Enhancement of a *Saccharum spontaneum* Population and a Genetic Impact Analysis of the Agronomic and Yield Traits of Its Progeny

**DOI:** 10.3390/plants14121750

**Published:** 2025-06-07

**Authors:** Jiayong Liu, Maoyong Ran, Liping Zhao, Lianan Tao, Fenggang Zan, Li Yao, Xin Hu, Shenglin Ren, Yong Zhao, Hongming Xia, Jing Zhang, Xinyuan Pu, Zhongfu Zhang, Zuhu Deng

**Affiliations:** 1 National Engineering Research Center for Sugarcane, Fujian Agriculture and Forestry University, Fuzhou 350002, China; 2 National Key Laboratory for Biological Breeding of Tropical Crops, Kunming 650205, China; 3 Sugarcane Research Institute, Yunnan Academy of Agricultural Sciences/Yunnan Key Laboratory of Sugarcane Genetic Improvement, Kaiyuan 661699, China

**Keywords:** *Saccharum spontaneum*, hybrid, sugarcane improvement, sugarcane breeding, genetic variation

## Abstract

*Saccharum spontaneum* serves as an essential genetic resource for sugarcane improvement. Traditional breeding methods, characterized by slow selection and limited germplasm exploitation, often lead to suboptimal progeny performance. In this study, we revised the utilization strategy by initially hybridizing several *S. spontaneum* clones, followed by intercrossing their F1 progeny to establish a heterogeneous ‘polymeric’ population, which was then subjected to ‘nobilization’. A natural *Saccharum spontaneum* (S0) plant was used as the parent to create a hybrid (S1) containing two *S. spontaneum* bloodlines. The agronomic traits of S1 were compared, leading to the identification of three superior hybrids. These hybrids were then crossed in a complete diallel design, resulting in six crosses. Significant genetic variation was observed for the agronomic traits. Compared with S0, the plant height in S1 increased by 31.5%, and by 32.22% in S2. The stem diameter in S1 increased by 38.71%, and by 51.61% in S2. The single stem weight increased by 125% in S1 and 150% in S2. Other yield traits also showed varying degrees of improvement. A correlation analysis indicated that the plant height, stalk diameter, single stalk weight, and leaf width were significantly positively correlated with yield, and the leaf width with brix. There was no significant correlation between the millable stalks and yield. This study successfully developed a novel *S. spontaneum* hybrid with significantly improved agronomic traits, enhancing the genetic foundation of *S. spontaneum* germplasm for nobilization breeding programs. These findings provide a valuable germplasm base for developing high-performance sugarcane varieties, improving the utilization of *S. spontaneum.*

## 1. Introduction

Sugarcane (*Saccharum* spp.) is the world’s primary source of sugar, accounting for over 80% of the global sugar production [1,2,3]. In China, more than 85% of sugar is derived from sugarcane [4]. The development and adoption of new sugarcane varieties are essential for the sustainable growth of the sugar industry. Hybrid breeding, the primary method for creating new sugarcane varieties, has significantly advanced the sugar industry.

Jeswiet’s theory of sugarcane nobilization represents a significant advancement in breeding practices [5]. This theory not only provided a framework for utilizing wild species of *S. spontaneum* in breeding programs but also accelerated the overall progress in sugarcane breeding [6]. The ‘nobilization’ process involves using the noble species *S. officinarum* as the female parent and *S. spontaneum* as the male parent. The resulting hybrid offspring serve as the male parent for subsequent backcrossing with *S. officinarum*. Due to the chromosome inheritance pattern of *2n + n* in the hybridization between *S. officinarum* and *S. spontaneum*, the chromosomes of *S. spontaneum* decrease rapidly, while the chromosomes of *S. officinarum* increase. This gene infiltration restores the high sucrose content characteristic of *S. officinarum*, while introducing the resistance traits of *S. spontaneum.* Among the wild species used in sugarcane hybridization, *S. spontaneum* is the most widely and effectively utilized [7]. Notable sugarcane varieties, such as the ‘first-generation sugarcane king’ POJ2878, have been bred from *S. spontaneum* [8]. However, the nobilization process is lengthy, with large offspring populations and each backcross relying on a single *S. spontaneum* clone. This results in a slow breeding process and limits the successful utilization of *S. spontaneum* germplasm for hybridization [9]. Reports indicate that only a limited number of *Saccharum spontaneum* clones have been successfully utilized in sugarcane breeding programs [10]. In 2020, the National Sugarcane Germplasm Resources Nursery of China collected 961 *S. spontaneum* resources, highlighting significant gaps in the development and utilization of *S. spontaneum* germplasm.

*S. spontaneum* is a highly valuable wild resource in sugarcane breeding; however, its full potential remains untapped due to limitations in current utilization methods. Traditionally, *S. spontaneum* have been used directly in hybridization, followed by the selection of agronomic traits. Nonetheless, undesirable traits such as a low sugar content and thin stalks persist [11,12]. To circumvent these issues, we implemented a germplasm enhancement strategy through targeted selection, producing elite hybrids with improved agronomic traits prior to noble hybridization. Previous studies have highlighted the genetic diversity within *S. spontaneum*, providing a solid theoretical and germplasm foundation for creating *S. spontaneum* hybrids [13,14]. By employing these hybrids in sugarcane nobilization, the efficiency of *S. spontaneum* resource utilization can be improved, and the breeding cycle shortened, offering superior parent materials for sugarcane breeding.

Heterosis allows for the integration of advantageous genes from both parents, resulting in offspring with enhanced traits [15,16,17]. This principle forms the theoretical basis for developing superior *S. spontaneum* materials in sugarcane breeding. The success of heterosis in crops like rice and maize has significantly advanced agricultural development [18,19,20]. In sugarcane, heterosis has been pivotal in improving yield, disease resistance, adaptability, and quality. This strategy also encompasses interspecific and intergeneric hybridization, along with backcrossing, to develop commercial cultivars. Thus, applying heterosis to enhance critical wild resources is essential in the sugarcane breeding process.

Current approaches to utilizing *S. spontaneum* in sugarcane breeding have limitations. Firstly, the use of heterosis to consolidate superior *S. spontaneum* genes is lacking. Secondly, breeding often involves a single *S. spontaneum* parent. Lastly, undesirable traits require multiple hybridizations to overcome, which lengthens breeding cycles. We propose the application of heterosis to enhance *S. spontaneum* before use in sugarcane breeding, thereby integrating superior genes and expediting the breeding cycle, advancing efforts in sugarcane improvement.

## 2. Materials and Methods

### 2.1. Materials

In 2018, three experimental populations were established through controlled crosses involving six original *Saccharum spontaneum* accessions at the Chinese National Sugarcane Germplasm Resource Nursery Kaiyuan. Hybridization procedures included emasculation of female parents through hot water treatment 50 °C for 5 min [21], with flower spikes subsequently enclosed in isolation bags to prevent pollen contamination. The resulting hybrid progeny Group A: YN82−1 × YN2017−22; Group B: VN2 × YN2017−12−165; and Group C: YN8 × YN2017−41 were cultivated in seedling greenhouses on 14 March 2019, yielding 35, 110, and 280 viable seedlings for Groups A, B, and C, respectively. Parental lines were concurrently propagated via stem bud cultivation on 10 April 2019. Field trials were conducted in Kaiyuan City, Yunnan Province, China, commencing on 11 June 2019. Experimental plots featured 1 m row spacing, 6 m row length, and 0.9 m plant spacing. Populations were arranged sequentially without replication, followed by their respective parental controls (two rows per parent). On 20 December 2019, elite S1 hybrids demonstrating superior phenotypic performance relative to parental lines were selected from each population (designated 2018-1/A1, 2018-2/B1, and 2018-3/C1). Quantitative traits including plant height, stem diameter, millable stalk count, leaf width, and brix percentage were recorded for both selected hybrids and parental lines (Table 1).

**Figure 1:**
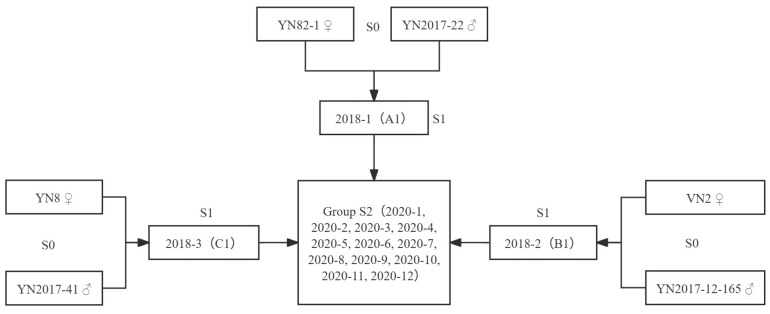
Material pedigree diagram.

Subsequently, A1, B1, and C1 served as parents for further hybridization, obtaining 42 progenies from the cross A1 × B1, 41 from A1 × C1, 38 from B1 × A1, 24 from B1 × C1, 45 from C1 × A1, and 2 from C1 × B1. Figure 1 illustrates the pedigree chart detailing the parentage of the materials. The resulting S2 progeny, along with the original S0 and S1 materials, underwent field cultivation for further analysis.

### 2.2. Preparation of Hybrid Crosses

In the initial stage of hybridization, clone plants from parent materials with strong growth and soil-covered bases at an appropriate height were selected. After photoperiod induction, those plants that had reached the booting stage but had not flowered, and exhibited robust root growth at the base were selected. These materials were transferred from the soil-covered base to a greenhouse environment. Plants were maintained in continuous-flow hydroponic systems to facilitate cross-pollination. Prior to pollination, the female parent underwent a hot water treatment at 50 °C to kill male pollen and prevent self-pollination. Specifically, a small incision was made with a surgical blade on the unbloomed female flower bud, which was then immersed in 50 °C water for 5 min. Sugarcane flower spikes designated for hybridization were placed in isolated cells to prevent cross-pollination [21].

### 2.3. Field Experiment

Experiment 1: This study employed two cultivation schemes: barrel planting and field cultivation. For barrel planting, seedlings Group S2 were placed in plastic buckets measuring 36.3 cm in diameter and 34 cm in height. Stem nodes from S0 and S1 generations were established as controls in identical cultivation barrel. To maintain soil consistency, soil was collected from barren mountains and was homogenized before placement in the buckets. The experiment was conducted in triplicate, with one seedling per bucket. Depending on the cross, 50 to 200 seedlings were planted, with the planting date was set for 25 June 2022. The aim was to conduct a preliminary screening of the S2 materials.

Experiment 2: For field cultivation, we utilized the materials screened in Experiment 1. This experiment was arranged in a randomized block design with three replicates. Each plot consisted of two rows, with each row measuring 6 m in length and spaced 1.1 m apart. Planting date was set for 7 June 2023. Subsequent management practices conducted in accordance with production standards. The site was located at an altitude of 1050 m, with an average annual rainfall of 800 mm and a mean temperature of 19.8 °C, positioned at 103°34′ E, 23°30′ N. Field management followed the established sugarcane production protocols of Kaiyuan City, Yunnan Province, China, encompassing fertilization, weed control, and integrated pest management practices. The objective was to systematically evaluate the performance of S2, S1, and S0 traits using screened materials, and to assess the genetic effects of key traits in *S. spontaneum.*

### 2.4. Statistical Analysis

Data were collected on plant height, stalk diameter, millable stalks, leaf width, and brix. Subsequently, calculations were performed for single stalk weight, cluster weight, millable stalks per hectare, and yield per hectare. The calculations are as follows:

Single stalk weight = plant height × stalk diameter^2^ × 0.785 (where 0.785 approximates 1/4π)/1000.

Cluster weight (kg) = single stalk weight (kg) × cluster millable stalks.

Millable stalks per hectare = millable stalks/m^2^ × 6667 m^2^ × 15.

Yield per hectare (kg/hectare) = single stalk weight (kg) × hectare millable stalks.

Mid-parent heterosis (MPH) and high-parent heterosis (HPH) were calculated using these formulas:

MPH = [(F1 − (P1 + P2)/2)**/**((P1 + P2)/2)] × 100%.

HPH = [(F1 − HP)/HP] × 100%, where HP indicates the highest trait value among parents.

Data statistical analysis was performed using Microsoft Excel 2019. Analysis of variance (ANOVA), correlation analysis, and hierarchical cluster analysis were conducted using SPSS Statistics version 21 [22,23,24]. Principal component analysis (PCA) and histogram visualization were carried out using R statistical software (version 4.4.3).

## 3. Results

### 3.1. Analysis of Agronomic Traits and Screening of Excellent Offspring Based on Data from Experiment 1

A variance analysis of key agronomic traits—including the plant height, stalk diameter, and brix—was performed on six diallel cross combinations, excluding selfing crosses. Significant differences were observed among the crosses for plant height (*p* = 0.047), stalk diameter (*p* = 0.007), millable stalks (*p* = 0.013), and brix (*p* = 0.01). No significant differences were found for cluster weight (*p* = 0.19) and leaf width (*p* = 0.35), as shown in Table 2.

To develop and identify the optimal ‘hybrid’ (S2) each deriving from four *S. spontaneum* clones for further research, we assessed the test materials using comprehensive performance metrics such as the stalk diameter, millable stalks, brix, and plant height, along with traits like the cluster weight. Six hybrid crosses were analyzed, using S0 and S1 as the controls. From the Experiment 1 results, 12 superior S2 plants were identified, as detailed in Table 3.

Compared to S0 and S1, the selected S2 materials demonstrated notable advantages in comprehensive traits, particularly against S0. It is important to highlight that while the overall performance is a key factor in the screening process, specific traits of certain materials are also critical selection criteria. For example, clone 2020-5 from the A1 × C1 cross showcased a superior stalk diameter, millable stalk count, and cluster weight, despite a lower brix compared to that of S1. Similarly, clone 2020-11 from the C1 × A1 cross exhibited significant millable stalk numbers and brix, even with its plant height and stalk diameter not matching S1’s.

We compared the average values of agronomic traits between two S1 materials and four S0 materials involved in the S2 generation. Despite certain traits of the S2 materials showing a downward trend compared to those of S1, the overall performance consistently exceeded that of both S1 and S0. For instance, the plant height and leaf width of the A1 × B1 progeny were reduced compared to the parental averages of A1 and B1, but the cluster weight, brix, and millable stalks were markedly improved over those of the parents, with all traits superior to those of S0. This might relate to the specific combining ability of the parents during hybridization. In order to more intuitively show the distribution of six important agronomic traits in each hybrid combination of the S2 population, we plotted a distribution histogram of the agronomic traits (Figures S1–S5). Due to the extremely low germination rate of C1 × B1, it was not possible to screen for good S2 progeny in this combination.

Based on the data presented in Table 3, the heterosis in the hybrid combinations was calculated. Mid-parent heterosis (MPH) and high-parent heterosis (HPH) demonstrated different ranges of variation (Table S1). In general, almost all of the traits exhibited lower HPH values compared to the MPH values. The range of MPH varied from −100% to 871%, whereas HPH varied from −74% to 580%. For all traits, both the MPH and HPH values were higher in S1 compared to S2.

### 3.2. Genetic Progress and Comparative Analysis of Hybrids Based on Data from Experiment 2

To further examine the genetic progress and advantages of the ‘hybrid’ in various traits, we conducted a comparative analysis of key agronomic traits among S0, S1, and S2. The findings revealed that the S1 group surpassed the S0 group across all traits, with significant differences (at the 0.05 level) observed in plant height, stalk diameter, leaf width, and single stalk weight. The yield differences were highly significant (*p* < 0.01), whereas no significant variation was detected in the brix content. Except for the millable stalk count, all the other traits of S2 were superior to those of S0 (Table 4). Although the millable stalks in S2 exhibited a declining trend, they remained high at 247,035 stalks/ha, showing no significant difference between S2 and S0. S2 demonstrated a significantly higher stalk diameter and single stalk weight than the original S. spontaneum (S0), with increases of 51.61% and 150.00%, respectively. These traits are crucial for the enhancement of wild germplasm. S2 also achieved the highest yield, 157.62% greater than S0, with an extremely significant difference (at the 0.01 level). The plant height and leaf width were also significantly improved compared to those of S0. This indicates that the development of ‘hybrids’ has substantially enhanced the production potential of S. spontaneum. Although brix, a quality trait, did not show a significant increase, S2 still exhibited an 8.90% relative increase compared to that of S0.

The top-performing clones from the S1 and S2 populations were systematically compared to evaluate the genetic progress (Table 5). Notably, the S2 clones demonstrated a superior agronomic performance, with clone 2020-7 exhibiting a 46.34% yield advantage over the highest-yielding S1 clone (2018-1). The brix levels showed incremental improvement, with 2020-7 exceeding the S1 maximum (2018-2) by 0.49 percentage points. While the millable stalk density of 2020-7 (378,030 stalks ha^−1^) remained 36.1% lower than the S1 maximum (591,919 stalks ha^−1^ in 2018-3), this was compensated for by substantial enhancements in stalk morphology. The stalk diameter of 2020-7 increased by 61.67% compared to 2018-3, concomitant with 266.67% and 95.88% increases in the single stalk weight and yield per hectare, respectively. Crucially, both 2020-7 and 2020-4 in S2 surpassed the S1 yield benchmark while maintaining a higher stalk density than the best S1 performer. These findings underscore significant genetic gains in the S2 germplasm, particularly in terms of yield component architecture optimization.

**Figure 2:**
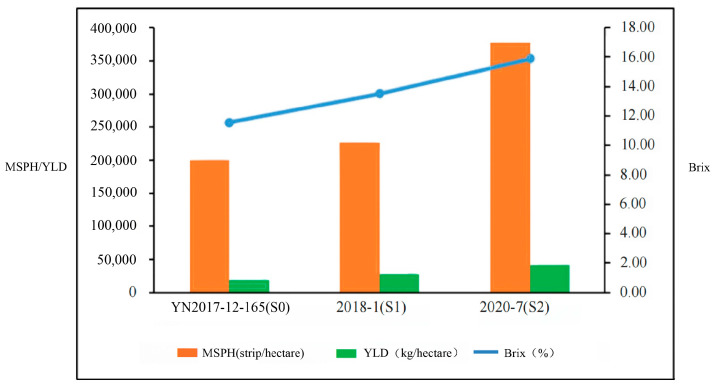
Comparison of germplasm differences with the best yield performance in S0, S1, and S2 groups in Experiment 2. MSPH = millable stalk per hectare, and YLD = yield per hectare.

A comparative analysis of the elite germplasms across the breeding cycles (S0–S2) revealed progressive trait improvements (Table 5, Figure 2). The S2 selection 2020-7 demonstrated composite trait superiority, exhibiting 46.34% and 125.5% yield increments relative to the S1 (2018-1) and S0 (YN 2017-2-165), respectively. Notably, the S1 progenitor 2018-1 itself showed a 54.10% yield enhancement over the S0 baseline. The brix value of 2020-7 reached 15.90%, representing 2.38 and 4.35 percentage point increases compared to the S1 and S0 controls, with parallel improvements in the millable stalk density (89.02% and 67.08% over respective checks). Through polygene pyramiding of four S. spontaneum parents, 2020-7 achieved concurrent optimization of yield components (MSPH = 378,030 strip/hectare) and quality parameters. These findings collectively demonstrate that strategic parent aggregation in S2 generated synergistic genetic gains across multiple agronomic traits.

**Figure 3:**
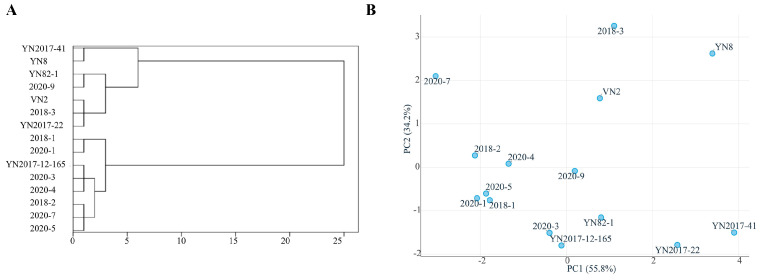
Hierarchical cluster analysis and principal component analysis of 15 materials from S0, S1, and S2 in Experiment 2. (**A**): cluster analysis; (**B**): principal component analysis.

The hierarchical cluster and principal component analyses were used, exploring eight specific traits—plant height, stalk diameter, millable stalks, brix, leaf width, single stalk weight, millable stalks per hectare, and yield per hectare, as listed in Table 5—in 15 varieties. As depicted in Figure 3A, these varieties are distinctly divided into two major groups. The first group, comprising seven varieties (five S0: YN 2017-41, YN 8, YN 82-1, VN 2, YN 2017-22; one S1: 2018-3; one S2: 2020-9), is characterized by a shorter plant height, slimmer stalks, a lighter single stalk weight, and a lower yield. In contrast, the eight varieties in the second group—including five S2: 2020-1, 2020-3, 2020-4, 2020-5, 2020-7; two S1: 2018-1, 2018-2; and one S0: YN2017-12-165—exhibit opposite traits. Notably, YN 2017-12-165 (S0) showcases the highest yield, as indicated in Figure 2, surpassing other S0 materials with its taller plant height, thicker stalks, and superior overall performance. Predominantly consisting of S1 and S2 varieties, this group underscores the significant genetic improvements in the hybrids as compared to the ancestral S. spontaneum (S0). In the principal component analysis (PCA), the first two principal components together accounted for 90% of the total variance in the dataset, effectively capturing the underlying distribution patterns of the samples. As shown in Figure 3B, the spatial arrangement of the samples in the PC1–PC2 biplot closely corresponded to the results of the hierarchical cluster analysis, thereby providing strong validation for the classification scheme derived from clustering. This concordance between multivariate analytical methods reinforces the reliability of the identified sample groupings.

### 3.3. Correlation Analysis Between Yield and Agronomic Traits

**Figure 4:**
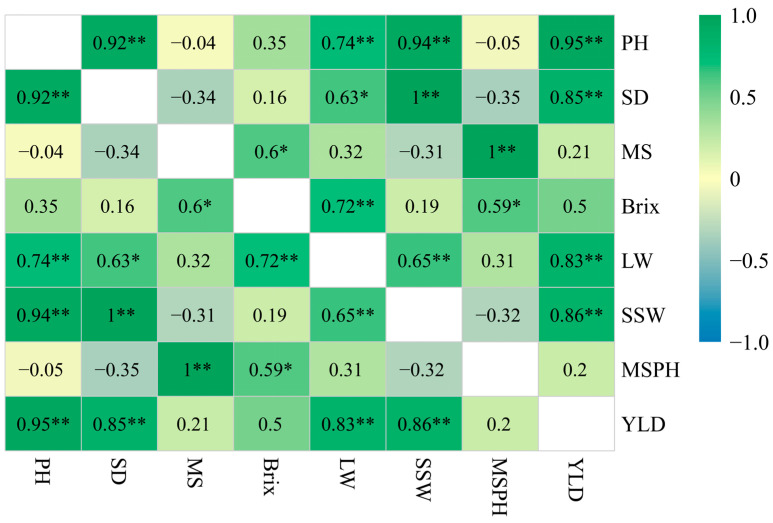
The correlation analysis between key agronomic traits and yield in Experiment 2. Note: PH = plant height, SD = stalk diameter, MS = millable stalks, LW = leaf width, SSW = single stalk weight, MSPH = millable stalks per hectare, and YLD = yield per hectare. The degree of correlation is color-coded according to the color key on the right. The blue and red boxes indicate negative and positive correlation coefficients, respectively. The symbols * and ** indicate significance at 0.05 and 0.01 probability, respectively.

A correlation analysis conducted on the average values of yield and other pivotal agronomic traits (Figure 4) revealed a substantial positive correlation between plant height and stalk diameter, and between single stalk weight and yield. No significant negative correlation was observed with the millable stalks per hectare. The stalk diameter was also positively correlated with both the single stalk weight and yield per hectare, whereas there was no significant negative correlation with the millable stalks per hectare. Additionally, the single stalk weight and yield per hectare exhibited a noteworthy positive correlation, while a negative correlation was noted between the single stalk weight and millable stalks per hectare. The correlation between the millable stalks per hectare and yield per hectare was marginal. Importantly, the plant height, stalk diameter, and single stalk weight were positively correlated with the yield per hectare. Conversely, the influence of the millable stalks per hectare on the yield was minimal, which can be attributed to its negative (though not significant) relationship with the aforementioned traits, reducing its impact on the yield. Moreover, the leaf width showed a significant positive correlation with both brix and plant height, suggesting that an increased area for light interception could enhance organic matter accumulation and biomass production.

## 4. Discussion

Germplasm resources are crucial for crop breeding. *S. spontaneum*, the most successful wild species utilized in sugarcane breeding, facilitated a significant advancement in the history of sugarcane development with the integration of its genome [11,25]. However, sugarcane breeding currently faces a bottleneck regarding the genetic enhancement of essential traits. Addressing this issue effectively necessitates the further development and utilization of germplasm resources. As one of the most invaluable wild species in the sugarcane genus complex, *S. spontaneum* stands as a prime candidate for focused breeding efforts.

### 4.1. The Application Status and Improvement Methods of S. spontaneum

*S. spontaneum* is notably diverse and abundant in nature [10]. Despite contributing 10 to 20 percent of the genetic background of modern sugarcane varieties, the potential of *S. spontaneum* to broaden the genetic base, enhance resistance, and improve regeneration remains largely untapped [26,27,28,29]. Current utilization methods do not maximize the breeding potential of *S. spontaneum.* Traditional methods are marred by lengthy breeding cycles, limited resource utilization, and the strong wild characteristics of *S. spontaneum*, which hinder effective cross-breeding selection. Consequently, there is a pressing need to innovate and refine breeding strategies for *S. spontaneum.* The genetic variability of agronomic traits is intricately linked to heterosis, a phenomenon where hybrid progeny exhibit superior traits compared to their parents, serving as a crucial mechanism for genetic improvement in crops. By enhancing parental lines’ agronomic traits, the heterosis of hybrid progeny can be effectively improved [30]. Proposed is a novel breeding strategy for *S. spontaneum*, utilizing original material as parents and employing cross-breeding techniques to acquire genetic materials with significantly enhanced agronomic traits, thereby advancing sugarcane breeding.

### 4.2. Correlation Effect

The interplay between agronomic traits, yield traits, and the overall yield is complex, posing challenges in achieving an optimal hybrid configuration [31,32]. Therefore, understanding the relationship between these traits and yield and optimizing hybrid combinations for maximal yield benefits is crucial [33]. This study revealed significant positive correlations between yield and several traits, including the single stem weight, plant height, stem diameter, and leaf width. The leaf width also showed significant positive correlations with the single stem weight, plant height, and brix, as well as with the stem diameter, suggesting its role in increasing the light capture area and enhancing photosynthesis, thereby boosting the nutrient supply for sugarcane growth. Typically, tropical sugarcane varieties exhibit broader leaves and a higher sugar content. Moreover, there was a significant positive correlation between the number of effective stems and brix, but a negative correlation with plant height, stem diameter, and single stem weight, which could be due to the reduced nutrient allocation per tiller when there are more tillers. *S. spontaneum*, known for its high tiller count and thin stems with a low sugar content, follows this pattern. Some *S. spontaneum* hybrids in this study demonstrated a reduction in the number of effective stems compared to their parent plants, with increased plant height, stem diameter, and brix. Subsequent parent selections might thus reduce the proportion of effective stems, and choosing those with larger single stem weights could also help lower labor costs during harvest.

### 4.3. Application of Improved S. spontaneum Progeny in Sugarcane Breeding

Germplasm resources are pivotal in crop breeding. *S. spontaneum*, a valuable wild species in sugarcane breeding, has made considerable contributions to enhancing various traits [33]. To foster genetic improvements in key sugarcane traits, it is imperative to exploit and develop germplasm resources such as *S. spontaneum* further. Despite the rich germplasm available and its essential role in breeding, no notable breakthrough varieties have emerged since the development of King POJ2878 [34]. This stagnation could be ascribed to the methodologies and efficiency in utilization rather than a scarcity of breeding potential. Presently, there are 1670 *S. spontaneum* clones preserved in China and the United States, yet only a marginalized number have been effectively utilized. This discrepancy underscores the inefficiencies in resource application. Addressing these challenges, this study introduces a novel strategy to enhance *S. spontaneum* resources by aggregating multiple pedigrees through population improvement, aiming to boost trait performance. The formation of such ‘aggregates’ opens new pathways for germplasm innovation in breeding, significantly enhancing the utilization efficiency and breeding success. With its extensive genetic diversity, *S. spontaneum* provides a robust theoretical basis for this methodology. By merging various *S. spontaneum* pedigrees, the method developed in this study holds promise for transcending traditional breeding limitations and elevating trait performance, thereby expediting genetic advances and setting the stage for future enhancements in sugarcane breeding.

This study has developed two *S. spontaneum* hybrids, referred to as S1 and S2, which have demonstrated exceptional traits. The remarkable performance of these hybrids underscores their potential to significantly advance sugarcane breeding by leveraging the genetic qualities of *S. spontaneum.* Furthermore, these findings facilitate ongoing hybridization efforts with *S. spontaneum*, encouraging continuous innovation and the development of superior hybrid strains. An important forthcoming research task involves employing cytological methods to explore if the *2n + n* chromosome inheritance pattern persists, and whether classical breeding theories remain relevant when these hybrids serve as the male parent in crosses with *S. officinarum*. Accomplishing this will further validate the practical utility of enhanced *S. spontaneum* in sugarcane breeding programs.

## 5. Conclusions

The comprehensive analysis of genetic variation in the hybrid offspring of *S. spontaneum* has confirmed the potential to enhance less desirable traits through selective breeding. The cross selection produced the S1 (A1, B1, C1) and S2 generations, which exhibit significant improvements over their parental lines in key agronomic and yield traits, thus enriching the germplasm resources for sugarcane breeding. Additionally, by comparing the agronomic traits between these hybrids, several superior offspring were identified. A correlation analysis between the agronomic traits and yield was also performed to elucidate the interdependencies between these factors. This study lays a theoretical foundation for strategic parent selection in future breeding endeavors. These insights offer a robust research basis for further enhancing the utilization of *S. spontaneum* in sugarcane breeding practices, aiming to foster significant advancements in the field.

## Figures and Tables

**Table 1:** Three S1 plants (2018-1, 2018-2, 2018-3) and their parents’ trait performance in Experiment 1.

Name of the Material	Classification	PH (cm)	SD (cm)	MS	LW (cm)	Brix (%)	CW (kg/Cluster)
YN82-1	Male of A1 (S0)	60	0.33	20	0.63	14.20	0.13
YN2017-22	Female of A1 (S0)	80	0.67	31	0.85	13.00	1.10
2018-1	A1 (S1)	130	1.20	32	1.47	15.60	5.99
VN2	Male of B1 (S0)	90	0.77	17	1.17	16.20	0.88
YN2017-12-165	Female of B1 (S0)	100	0.90	16	0.83	13.20	1.32
2018-2	B1 (S1)	140	1.13	21	2.03	17.40	3.78
YN8	Male of C1 (S0)	50	0.55	25	0.93	15.00	0.37
YN2017-41	Female of C1 (S0)	60	0.50	21	0.67	14.20	0.31
2018-3	C1 (S1)	80	0.87	47	1.47	17.00	2.82

Note: YN = Yunnan, VN = Vietnam, PH = plant height, SD = stalk diameter, MS = millable stalk, LW = leaf width, and CW = cluster weight.

**Table 2:** The mean and variance analysis of characters of the crosses in Experiment 1.

Trait	Combined Mean						*p*
	A1 × B1	A1 × C1	B1 × A1	B1 × C1	C1 × A1	C1 × B1	
PH (cm)	168	172	148	170	171	159	0.047
SD (cm)	1.10	1.10	0.90	0.90	1.00	0.90	0.007
MS (stalk/cluster)	12	13	10	20	17	25	0.013
Brix (%)	7.70	7.70	6.80	7.00	7.30	5.70	0.010
CW (kg)	3.70	4.30	2.00	4.60	4.60	5.06	0.190
LW (cm)	1.70	1.80	1.80	1.80	1.90	1.58	0.350

Note: *p* < 0.05 indicates a significant difference, while *p* < 0.01 indicates an extremely significant difference. PH = plant height, SD = stalk diameter, MS = millable stalks, CW = cluster weight, and LW = leaf width.

**Table 3:** The average value of 6 traits of 12 excellent S2 progeny and related parents in Experiment 1.

Name of the Material	Cross Source	PH (cm)	SD (cm)	MS (Stalk/Cluster)	Brix (%)	CW (kg)	LW (cm)	Type
2020-1	A1 × B1	202	1.2	17	8.2	3.97	1.6	S2
2020-2	A1 × B1	205	1.2	13	7.3	3.18	1.8	S2
2020-3	A1 × B1	197	0.9	24	9.3	3.23	1.6	S2
Mean of A1 and B1		211	1.1	11	8.3	2.20	2.1	S1
Mean of parent of A1 and B1		176	0.8	14	8.3	1.24	1.5	S0
2020-4	A1 × C1	207	1.1	13	9.4	2.60	1.7	S2
2020-5	A1 × C1	197	1.2	25	7.8	5.25	2.0	S2
Mean of A1 and C1		212	1.0	12	8.7	2.00	2.0	S1
Mean of parent of A1amd C1		150	0.5	15	6.7	0.44	1.5	S0
2020-6	B1 × A1	159	1.0	26	6.3	3.18	2.0	S2
Mean of B1 and A1		211	1.1	11	8.3	2.20	2.1	S1
Mean of parent of B1 and A1		176	0.8	14	8.3	1.24	1.5	S0
2020-7	B1 × C1	188	0.9	42	10.1	4.60	2.4	S2
2020-8	B1 × C1	157	0.9	30	8.6	3.17	1.4	S2
2020-9	B1 × C1	212	0.8	24	10.5	2.50	2.0	S2
Mean of B1 and C1		207	0.9	18	8.3	2.30	2.1	S1
Mean of parent of B1 and C1		161	0.6	14	8.0	0.61	1.5	S0
2020-10	C1 × A1	229	1.2	12	9.2	2.82	2.3	S2
2020-11	C1 × A1	161	0.9	29	12.9	2.92	1.4	S2
2020-12	C1 × A1	168	1.0	17	8.9	2.32	1.4	S2
Mean of C1 and A1		212	1.0	12	8.7	2.00	2.0	S1
Mean of parent of C1 and A1		150	0.5	15	6.7	0.44	1.5	S0
A1	YN82-1 × YN2017-22	216	1.2	6	8.8	1.55	1.9	S1
B1	VN2 × YN2017-12-165	206	1.0	17	7.9	2.85	2.3	S1
C1	YN8 × YN2017-41	208	0.7	19	8.7	1.70	2.0	S1
YN2017-12-165		193	1.1	9	7.5	1.65	1.2	S0
YN2017-22		138	0.6	23	6.0	1.00	1.5	S0
YN2017-41		115	0.4	10	7.1	0.10	1.2	S0
VN2		183	0.7	17	11.8	1.10	1.8	S0
YN8		156	0.3	20	5.7	0.25	1.7	S0
YN82-1		193	0.9	8	7.8	1.10	1.5	S0

Note: PH = plant height, SD = stalk diameter, MS = millable stalk, CW = cluster weight, and LW = leaf width. YN = Yunnan, and VN = Vietnam. The mean of A1 and B1 represents the overall average values of parent A1 and parent B1. The mean of parent of A1 and B1 represents the overall average values of parent (2017-22, 82-1) of A1 and parent (Vietnam 2, Yunnan 2017-12-165) of B1, respectively. The same notation applies similarly throughout.

**Table 4:** The mean of each trait in S0 group, S1 group and S2 group in Experiment 2.

Trait	S0 (CK)	S1	Increase Compared with S0 %	S2	Increase Compared with S0 %
PH (cm)	107.33	141.14 *	31.50	141.91 *	32.22
SD (cm)	0.62	0.86 *	38.71	0.94 **	51.61
MS	18.73	23.27	24.24	16.30	−12.97
Brix (%)	13.15	14.66	11.48	14.32	8.90
LW (cm)	0.93	1.32 *	41.94	1.37 *	47.31
SSW (kg)	0.04	0.09 *	125.00	0.10 **	150.00
MSPH	283,830	352,560	24.24	247,035	−12.97
YPH (kg)	9695.55	24,821.25 **	156.01	24,977.70 **	157.62

Note: The S0 group includes all the original S. spontaneum individuals, the S1 group includes all the S1 individuals, and the S2 group includes all the S2 individuals. A single asterisk (*) indicates a significant difference compared to S0, while a double asterisk (**) indicates an extremely significant difference compared to S0. PH = plant height, SD = stalk diameter, MS = millable stalk, BX = brix, LW = leaf width, SSW = single stalk weight, MSPH = millable stalk per hectare, and YPH = yield per hectare.

**Table 5:** The mean of 8 traits of the ‘hybrid’ (S1 and S2) and its parent in Experiment 2.

Name	Type	PH (cm)	SD (cm)	MS	Brix (%)	LW (cm)	SSW (kg)	MSPH (strip/hectare)	YLD (kg/hectare)
YN2017-12-165	S0	142	0.90	13	11.55	0.97	0.09	199,995	18,186.00
YN2017-22	S0	98	0.59	12	11.42	0.88	0.03	180,300	4847.25
YN2017-41	S0	66	0.42	11	12.65	0.67	0.01	173,220	1646.25
VN2	S0	126	0.63	25	16.00	1.14	0.04	373,095	14,829.15
YN8	S0	85	0.40	38	13.89	1.01	0.01	573,780	6201.00
YN82-1	S0	124	0.79	13	13.36	0.90	0.06	202,575	12,463.95
2018-1	S1	145	1.04	15	13.52	1.25	0.12	226,260	28,020.90
2018-2	S1	153	0.95	16	15.41	1.52	0.11	239,520	25,509.15
2018-3	S1	124	0.60	39	15.04	1.19	0.03	591,915	20,933.85
2020-1	S2	144	1.06	14	14.08	1.35	0.13	204,915	26,401.50
2020-3	S2	141	0.92	12	12.75	1.03	0.09	184,725	17,762.70
2020-4	S2	140	0.95	18	14.44	1.25	0.10	279,165	27,625.20
2020-5	S2	151	0.99	14	14.09	1.32	0.12	219,180	25,460.70
2020-7	S2	147	0.97	25	15.90	1.88	0.11	378,030	41,005.95
2020-9	S2	125	0.74	14	14.69	1.42	0.05	216,150	11,610.30

Note: PH = plant height, SD = stalk diameter, MS = millable stalk, LW = leaf width, SSW = single stalk weight, MSPH = millable stalk per hectare, YLD = yield per hectare, YN = Yunnan, and VN = Vietnam.

## Data Availability

The datasets supporting the conclusions of this manuscript and materials generated in this study are available from the corresponding author upon request.

## Supplementary Materials

The following supporting information can be downloaded at: https://www.mdpi.com/article/10.3390/plants14121750/s1.
