# Supplementary material for: The Enhancement of a Saccharum spontaneum Population and a Genetic Impact Analysis of the Agronomic and Yield Traits of Its Progeny
Source: Plants (Basel). 2025 Jun 7;14(12):1750. doi: 10.3390/plants14121750 (PMC12196922; doi:10.3390/plants14121750)
Supplement: Supplementary file 1 [file plants-14-01750-s001.zip › Supplementary Figure S1.pdf]

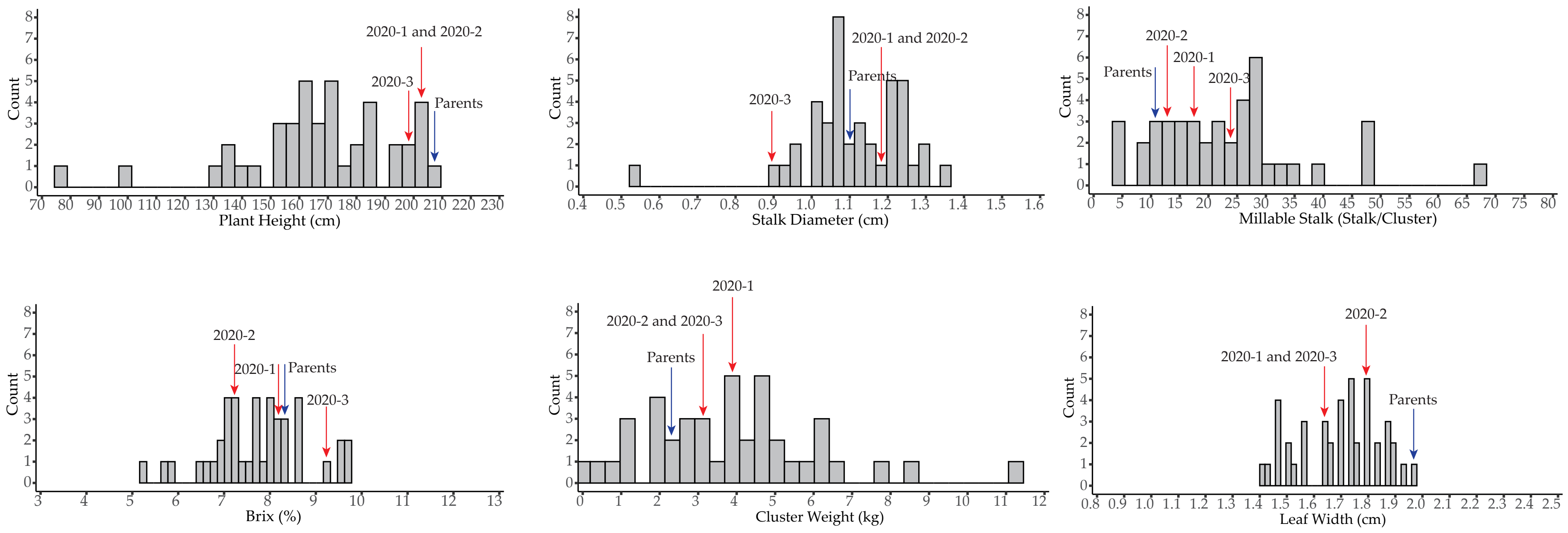

Supplementary Figure S1: Phenotypic distribution of six important agronomic traits in A1 × Bl hybrid progeny population, y-axis represents the number of plants, the red arrow represents the data of the selected S2, and the blue arrow represents the average value of the parent S1. Data correspond to Table 3 in the manuscript.

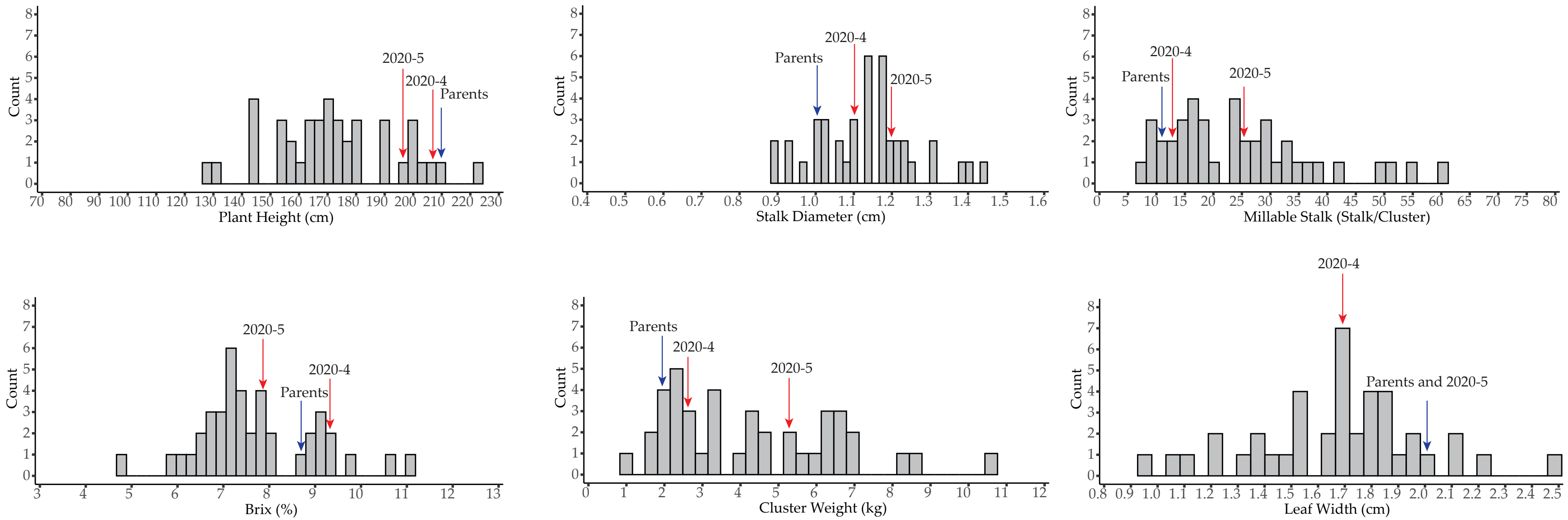

Supplementary Figure S1: Phenotypic distribution of six important agronomic traits in A1 × Cl hybrid progeny population, y-axis represents the number of plants, the red arrow represents the data of the selected S2, and the blue arrow represents the average value of the parent S1. Data correspond to Table 3 in the manuscript.

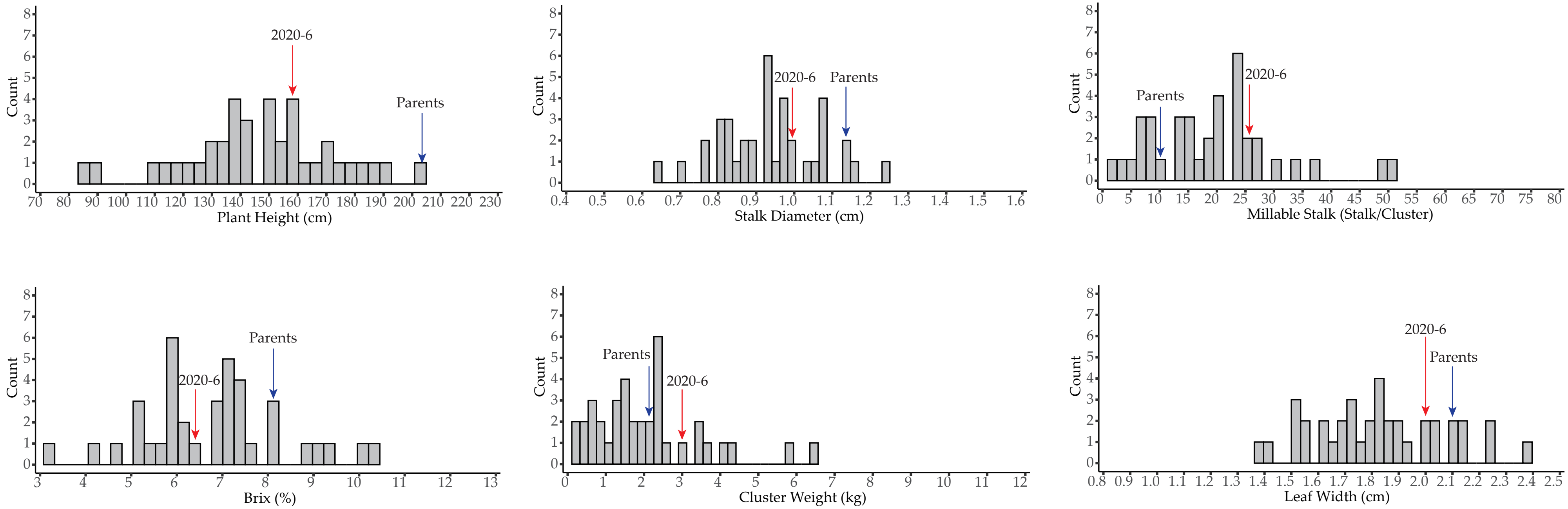

Supplementary Figure S1: Phenotypic distribution of six important agronomic traits in B1 × Al hybrid progeny population, y-axis represents the number of plants, the red arrow represents the data of the selected S2, and the blue arrow represents the average value of the parent S1. Data correspond to Table 3 in the manuscript.

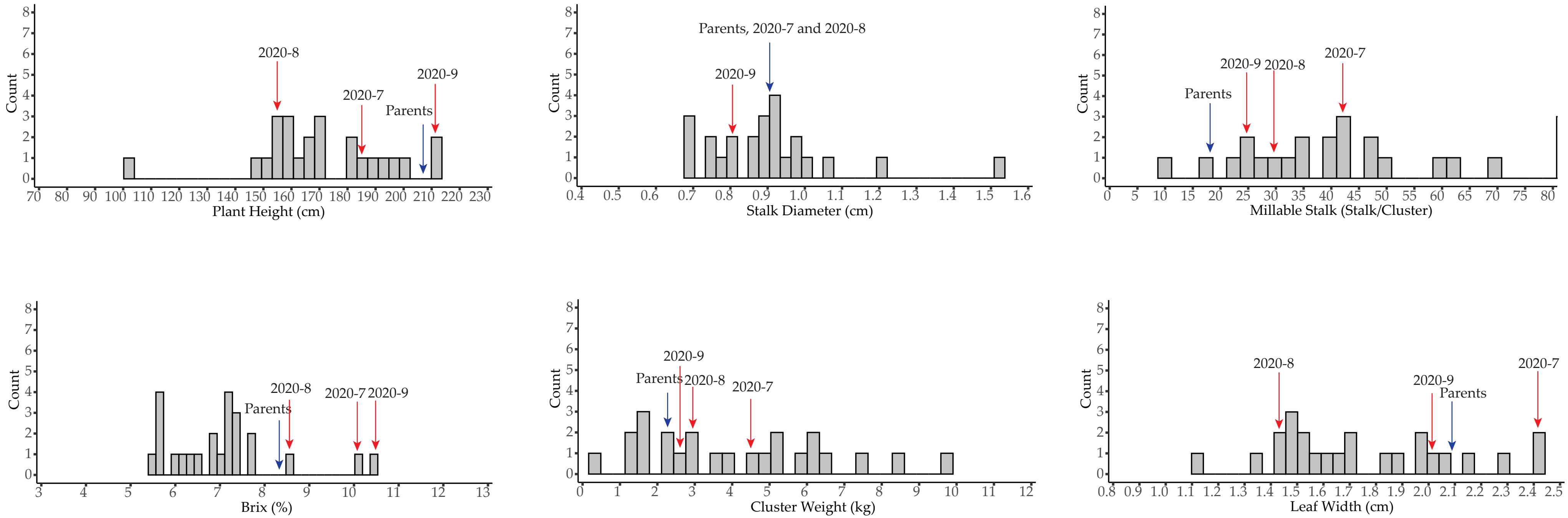

Supplementary Figure S1: Phenotypic distribution of six important agronomic traits in B1 × Cl hybrid progeny population, y-axis represents the number of plants, the red arrow represents the data of the selected S2, and the blue arrow represents the average value of the parent S1. Data correspond to Table 3 in the manuscript.

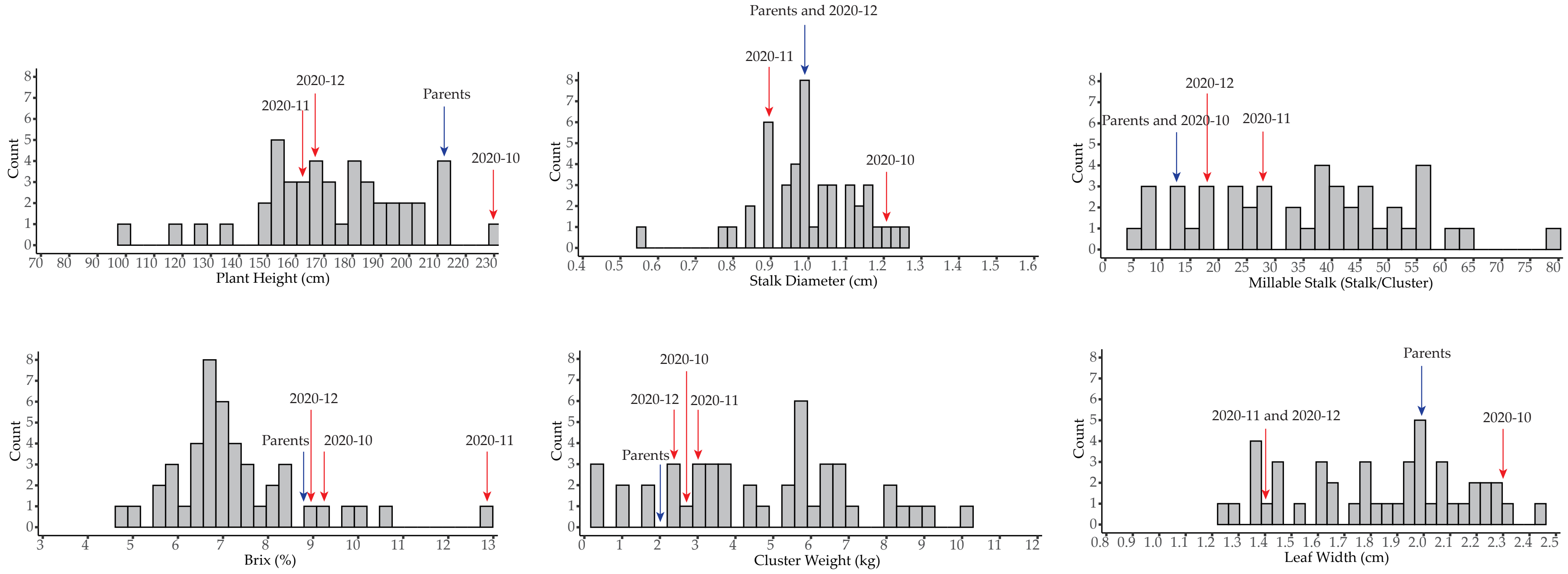

Supplementary Figure S1: Phenotypic distribution of six important agronomic traits in C1 × Al hybrid progeny population, y-axis represents the number of plants, the red arrow represents the data of the selected S2, and the blue arrow represents the average value of the parent S1. Data correspond to Table 3 in the manuscript.
