# Supplementary material for: The Enhancement of a Saccharum spontaneum Population and a Genetic Impact Analysis of the Agronomic and Yield Traits of Its Progeny
Source: Plants (Basel). 2025 Jun 7;14(12):1750. doi: 10.3390/plants14121750 (PMC12196922; doi:10.3390/plants14121750)
Supplement: Supplementary file 1 [file plants-14-01750-s001.zip › Table S1.pdf]

Table S1. Three S1 and 12 excellent S2 heterosis

name of the material	Cross Source	MPH						HPH					
		PH(%)	SD(%)	MS(%)	Brix(%)	CW(%)	LW(%)	PH(%)	SD(%)	MS(%)	Brix(%)	CW(%)	LW(%)
2020-1	A1×B1	-0.04	0.09	0.55	-0.01	0.80	-0.24	-0.06	0.00	0.00	-0.07	0.39	-0.30
2020-2	A1×B1	-0.03	0.09	0.18	-0.12	0.45	-0.14	-0.05	0.00	-0.24	-0.17	0.12	-0.22
2020-3	A1×B1	-0.07	-0.18	1.18	0.12	0.47	-0.24	-0.09	-0.25	0.41	0.06	0.13	-0.30
2020-4	A1×C1	-0.02	0.10	0.08	0.08	0.30	-0.15	-0.04	-0.08	-0.32	0.07	0.53	-0.15
2020-5	A1×C1	-0.07	0.20	1.08	-0.10	1.63	0.00	-0.09	0.00	0.32	-0.11	2.09	0.00
2020-6	B1×A1	-0.25	-0.09	1.36	-1.00	0.45	-0.05	-0.26	-0.17	0.53	-0.28	0.12	-0.13
2020-7	B1×C1	-0.09	0.00	1.33	0.22	1.00	0.14	-0.10	-0.10	1.21	0.16	0.61	0.04
2020-8	B1×C1	-0.24	0.00	0.67	0.04	0.38	-0.33	-0.25	-0.10	0.58	-0.01	0.11	-0.39
2020-9	B1×C1	0.02	-0.11	0.33	0.27	0.09	-0.05	0.02	-0.20	0.26	0.21	-0.12	-0.13
2020-10	C1×A1	0.08	0.20	0.00	0.06	0.41	0.15	0.06	0.00	-0.37	0.05	0.66	0.15
2020-11	C1×A1	-0.24	-0.10	1.42	0.48	0.46	-0.30	-0.25	-0.25	0.53	0.47	0.72	-0.30
2020-12	C1×A1	-0.21	0.00	0.42	0.02	0.16	-0.30	-0.22	-0.17	-0.11	0.01	0.36	-0.30
A1	YN82-1×YN2017-22	0.31	0.60	-0.61	0.28	0.48	0.27	0.12	0.33	-0.74	0.13	0.41	0.27
B1	VN2×YN2017-12-165	0.10	0.11	0.31	-0.18	1.07	0.53	0.07	-0.09	0.00	-0.33	0.73	0.28
C1	YN8×YN2017-41	0.54	1.00	0.27	0.36	8.71	0.38	0.33	0.75	-0.05	0.23	5.80	0.18
